# Lipid conformational order and the etiology of cataract and dry eye

**DOI:** 10.1194/jlr.TR120000874

**Published:** 2021-02-06

**Authors:** Douglas Borchman

**Affiliations:** 1 Department of Ophthalmology and Visual Sciences, University of Louisville, Louisville, KY 40202

**Keywords:** meibum, lens, membrane, spectroscopy, BR, blink rate, CE, cholesteryl ester, MHSCT, meibum from donors with dry eye due to hematopoietic stem cell transplantation, M_MGD_, meibum from donors with dry eye due to Meibomian gland dysfunction, M_n_, meibum from donors without dry eye, TBUT, tear breakup time, TFLL, tear film lipid layer, WE, wax ester

## Abstract

Lens and tear film lipids are as unique as the systems they reside in. The major lipid of the human lens is dihydrosphingomylein, found in quantity only in the lens. The lens contains a cholesterol to phospholipid molar ratio as high as 10:1, more than anywhere else in the body. Lens lipids contribute to maintaining lens clarity, and alterations in lens lipid composition due to age are likely to contribute to cataract. Lens lipid composition reflects adaptations to the unique characteristics of the lens: no turnover of lens lipids or proteins; the lowest amount of oxygen of any tissue; and contains almost no intracellular organelles. The tear film lipid layer (TFLL) is also unique. The TFLL is a thin (100 nm) layer of lipid on the surface of tears covering the cornea that contributes to tear film stability. The major lipids of the TFLL are wax esters and cholesterol esters that are not found in the lens. The hydrocarbon chains associated with the esters are longer than those found anywhere else in the body (as long as 32 carbons), and many are branched. Changes in the composition and structure of the 30,000 different moieties of TFLL contribute to the instability of tears. The focus of the current review is how spectroscopy has been used to elucidate the relationships between lipid composition, conformational order and function, and the etiology of cataract and dry eye.

The focus of the current review is how spectroscopy has been used to elucidate the relationships between lipid composition, conformational order and function, and the etiology of cataract and dry eye (keratoconjunctivitis sicca). Cataracts are a major cause of progressive irreversible blindness, particularly in underdeveloped countries, afflicting over 20 million people (1); but, unlike most other blinding eye diseases that are progressive, age-related, and irreversible, there is an easy corrective treatment for cataracts: cataract extraction and insertion of a synthetic intraocular lens. Dry eye is the major reason worldwide for seeking medical help and affects 5–50% of people worldwide, especially Asians (2). Since the last review of lens lipids and the etiology of cataracts in this journal a decade ago (3), insightful spectroscopic studies have been published suggesting a need for this review. Although numerous reviews related to the composition of tear lipids and the etiology of dry eye have been published recently (4, 5, 6, 7, 8, 9, 10, 11, 12, 13, 14, 15, 16), this is the first review article related to publications that use spectroscopic techniques such as infrared, NMR, fluorescence, and Brewster’s angle spectroscopies to elucidate relationships between tear lipid hydrocarbon chain conformational order and function related to the etiology of dry eye. Human lens lipid composition is unique, as the major phospholipid is dihydrosphingomyelin, found at high concentration (47%) only in human lenses, and it has an unusually high cholesterol to phospholipid molar ratio of 2:9 (Table 1). Tear lipids are also unique, as the predominant lipids are wax esters (WEs) and cholesteryl esters (CEs) (80%), with branched and very long hydrocarbon chains (Table 1). Whereas phospholipids make up most of the lens lipids, they only make up about 6% of the lipids found in tears. The relationships of these unusual lipids with structure and function form the basis of this review.

**Table 1:** Major lipids of the human lens and tear film

Lipid Species	Major Human Lens Lipids (17)	Major Human Tear Film Lipids (18)
Dihydrosphingomyelin^a^	47	0
Sphingomyelin^a^	19	18.3
Phosphatidylcholine^a^	11	30.2
Phosphatidylethanolamine (1-*O*-alkyl ether)^a^	15	0
Phosphatidylethanolamine^a^	0	8.5
Phosphatidylserine^a^	8	1.3
Phosphatidylinositol^a^	1	8.8
Phosphatidic acid^a^	0	0.9
Lyso phosphatidylethanolamine^a^	0	15.8
Lyso phosphatidylcholine^a^	0	6.0
Lyso phosphatidylserine^a^	0	5.6
Ceramide^a^	0	3.2
Cholesterol^b^	200% (equatorial), 900% (nucleus) (19, 20)	6
Phospholipids^b^	100	8.2
CEs^b^	0	44.8
WEs^b^	0	35.2
Triacylglycerides^b^	0	2.8
Diacylglycerides^b^	0	0.3
O-acyl-ω-hydroxy-fatty acid^b^	0	2.5

a Values are molar percent of phospholipids.

b Values are molar percent of all lipids.

## Function and unique characteristics of the lens

**Fig. 1:**
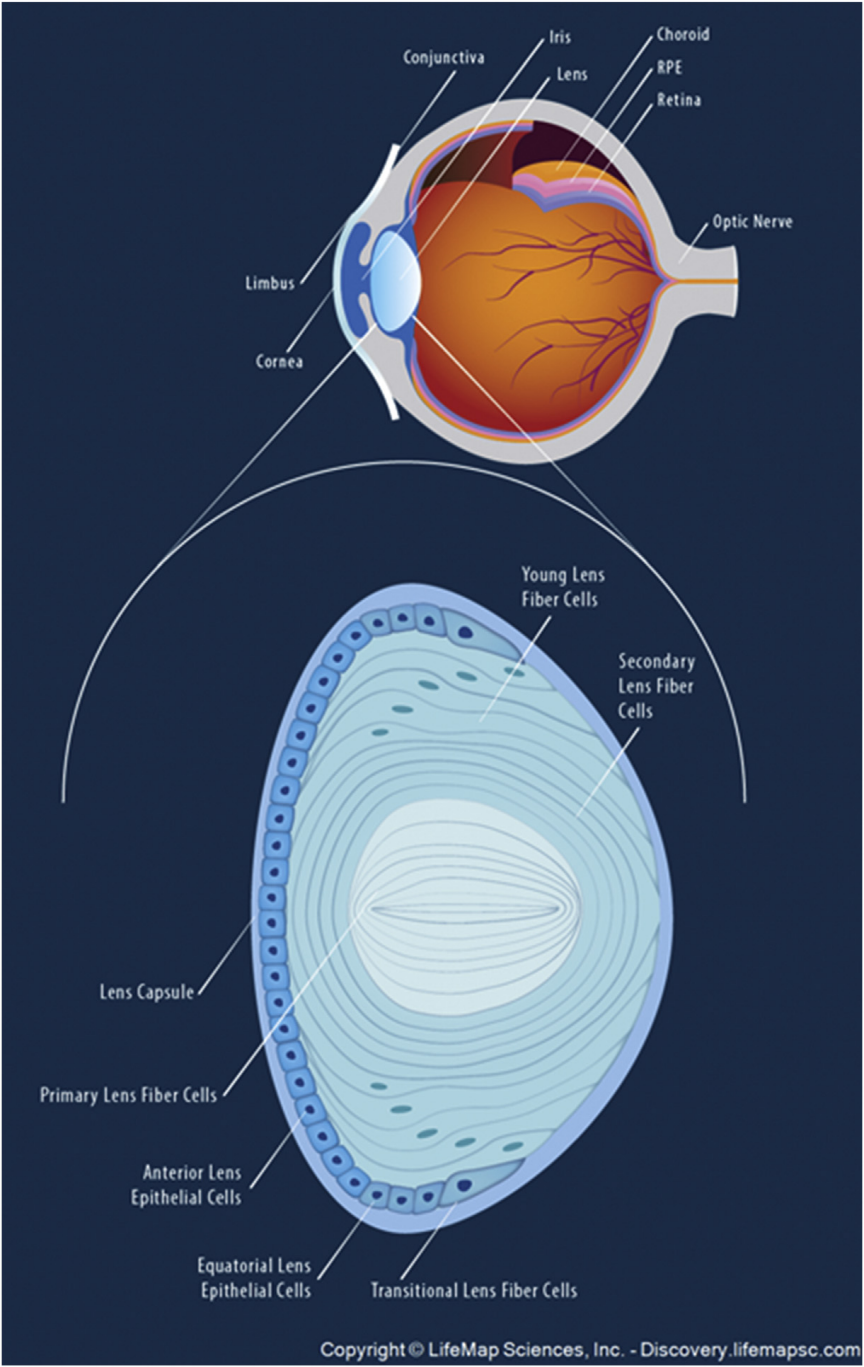
Top: Cross-section through a human eye. Bottom: Schematic of the human lens. Used with permission from LifeMap Sciences, Inc. (https://discovery.lifemapsc.com).

The purpose of the lens is to focus light onto the back of the eye (retina) where the light is transduced into an electric signal and is then interpreted by the brain as a visual image. Zonules attached to the equatorial region of the lens capsule surrounding the lens are attached to ciliary muscles that control zonular tension and change the shape and focus of the lens (Fig. 1). In order to be and remain clear, the lens is unique: it has no blood supply and thus, less oxygen than other organs (21); all of the cells are arranged in a crystalline hexagonal array; the space between the cells is smaller than the wavelength of light to avoid scattering; there are almost no intracellular organelles; and all of the biomolecules, such as the crystalline proteins, are arranged in a symmetrical crystalline array (22, 23). It is remarkable and yet unexplained how the lens adapted all of the features above, the absence of any one of which would cause the lens to become opaque and useless. The lens contains a thin monolayer of epithelial cells on the posterior surface that contains organelles (Fig. 1). The epithelial cells differentiate and elongate at the equator into fiber cells that are centimeters long, which, over time, migrate toward the center of the lens (24). As there is no turnover of lipids (25) and proteins (26) due to the lack of intracellular organelles (27, 28), with time, the lens increases in size and weight and many lens lipids and proteins are as old as the individual.

## Function and unique characteristics of the tear film lipid layer

**Fig. 2:**
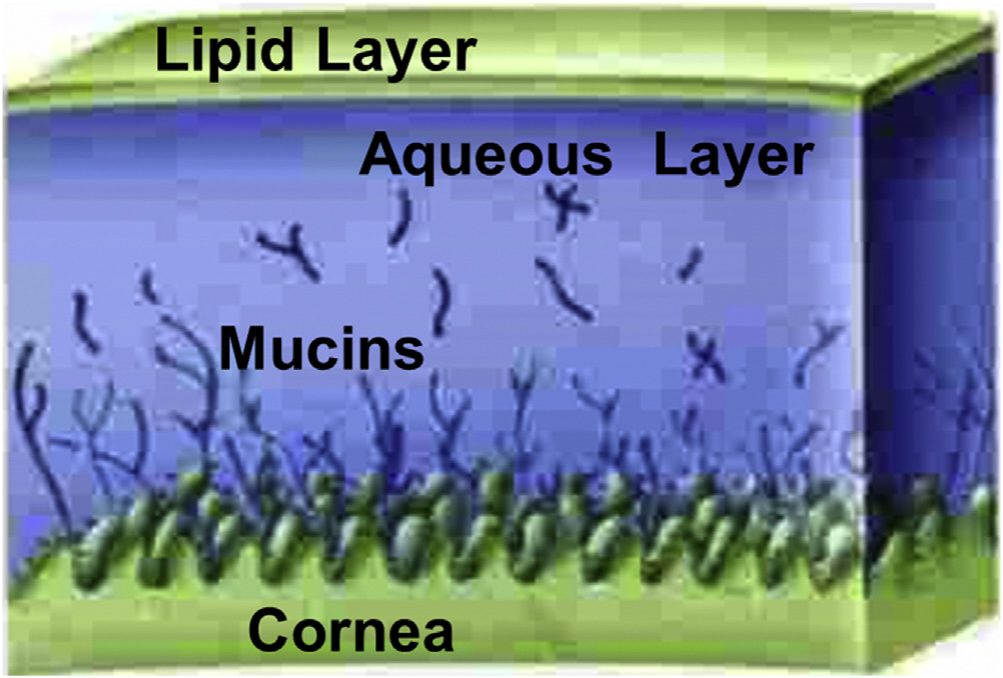
Schematic of the TFLL on the surface of the cornea adapted from slideshare.net (https://www.pharmaccutical-journal.com).

**Fig. 3:**
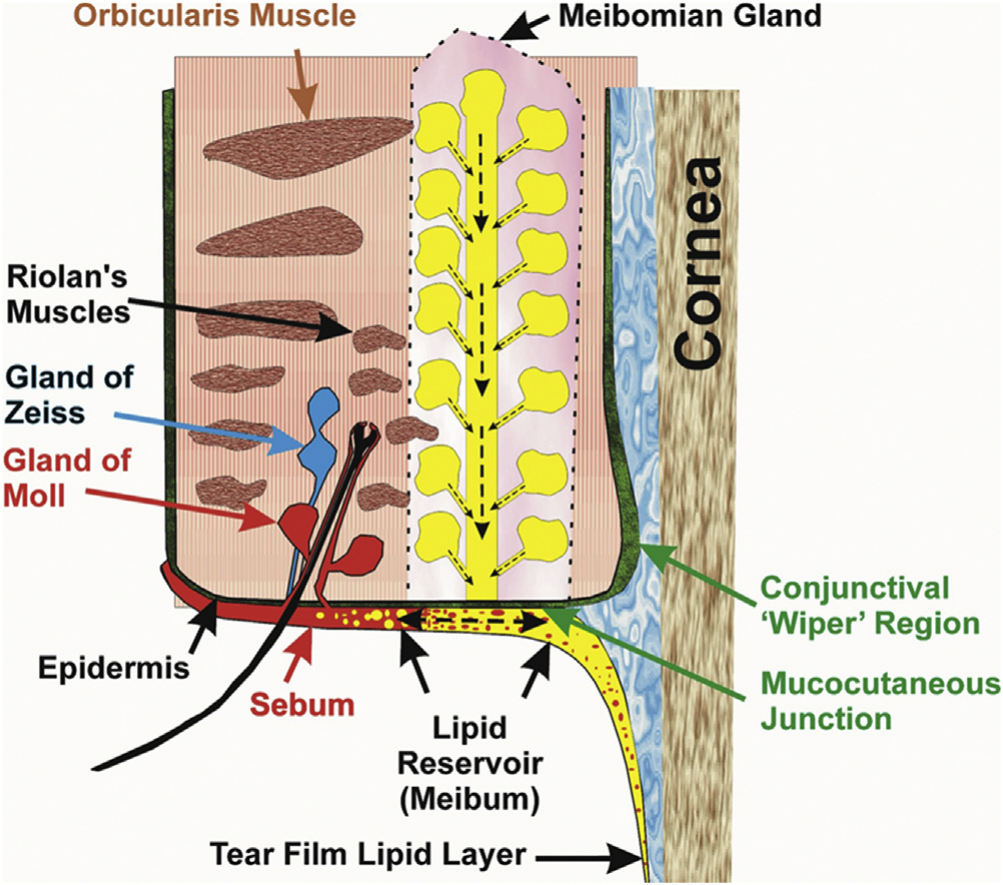
Cross-section through the eyelid. Sebum (red) is shown mixing with meibum (yellow), which forms a continuous TFLL film over the ocular surface and eyelid. From (32).

The tear film lipid layer (TFLL) is a thin (100 nm) lipid layer on the surface of tears (29, 30) covering the cornea that is 80 times thinner than the aqueous tear layer below (Fig. 2). The major source of the TFLL is the Meibomian gland that contributes about 80% of the TFLL (Fig. 3) (31, 32, 33, 34, 35). Meibomian glands (also known as “tarsal glands”) are named after Heinrich Meibom, a German physician who first described them; hence, the lipid secreted from the Meibomian gland is called “meibom” (8). It is speculated that some of the TFLL comes from the sebaceous glands in the eyelid (31) and lipid bound to lipocalin (35) originating from the lacrimal gland. The Meibomian gland is a sebaceous gland consisting of acini cells that constantly fill the gland with lipids. Upon blinking, which involves the contraction of the orbicularis and Riolan’s muscles, a small amount of lipid is squeezed out of the Meibomian glands onto the tear film surface. Thus, unlike the lens discussed above in which there is no turnover of lipid, fresh lipid is layered onto the tear film surface as often as one blinks. Upon blinking, the TFLL is drawn upward and the tear film spreads, driven by the Marangoni effect (15, 36, 37, 38). Shortly after blinking, which occurs about every 10 s, tears break up and the process starts over again with another blink. Thus, the major function of the TFLL is to aid in the spreading of tears. Other functions of the TFLL are to dam, lubricate, and stabilize the tear film to allow for proper refraction, to degrade mucinic clots, to provide an antibacterial effect, and to suppress exposure to UV rays (8).

## Structure/conformation of lens membranes

**Fig. 4:**
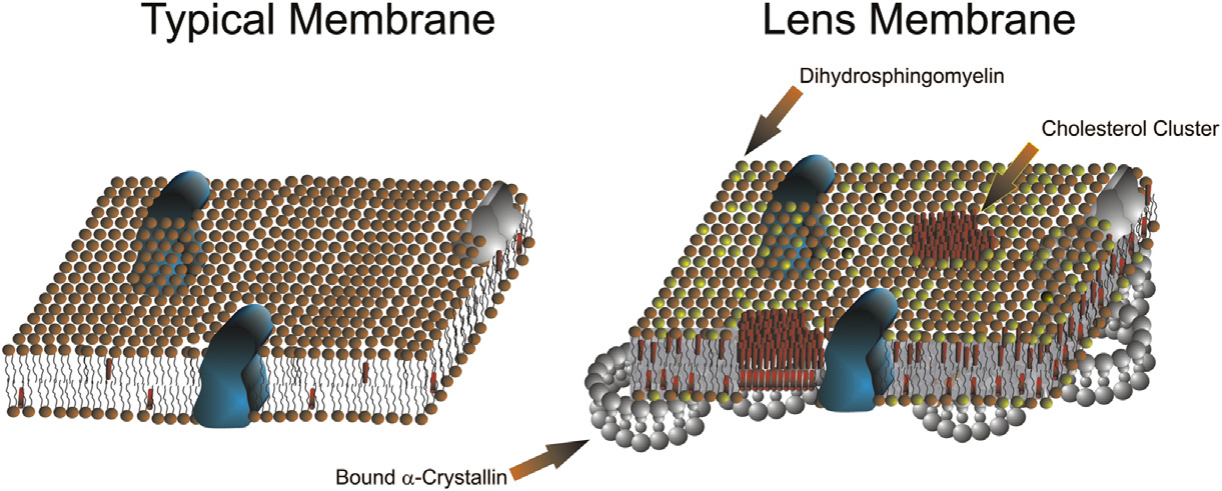
Left: A typical membrane. Right: Human lens membrane. Typical membranes contain fluid lipids with relatively few cholesterol molecules (red cylinders). Human lens membranes are unique. Most of the lipid is associated with proteins such as α-crystallin (α-crystallin assembly shown as gray balls, one large ball and one small ball for each α-crystallin) and aquaporin, which limits their mobility. Human lens membranes are some of the most saturated ordered (stiff) membranes in the human body. The major lipid of the human lens is dihydrosphingomyelin (green shaded balls). Found in quantity only in the human lens. From (3).

In passing through the human lens, light traverses through thousands of cellular membranes that scatter most of the light passing through the lens (3, 13, 39, 40). The amount of light scattered by lens membranes is related to lipid structural order or stiffness with age, cataract, and species (3). Lens membranes are important to the clarity of the lens, as membrane proteins such as aquaporin, plasma membrane Ca^2+^-ATPase, and Na,K-ATPase reside in the lens membranes of the epithelium and equatorial fibers and are necessary for maintaining the homeostasis of lens water, calcium, sodium, and potassium [references in (3, 13)]. Typical membranes are similar to the Singer fluid-mosaic model in which proteins float in a sea of fluid phospholipids with lateral mobility within the bilayer (Fig. 4, left) (41). Lens membranes are atypical, as membranes of adult human lenses are some of the most saturated and ordered membranes in the human body, and their high level of cholesterol leads to the formation of patches of pure cholesterol bilayers (Fig. 4, right) (18, 32). Furthermore, most of the lipids are associated with crystalline and membrane proteins, thus limiting their mobility (Fig. 4, right) (3, 13).

**Fig. 5:**
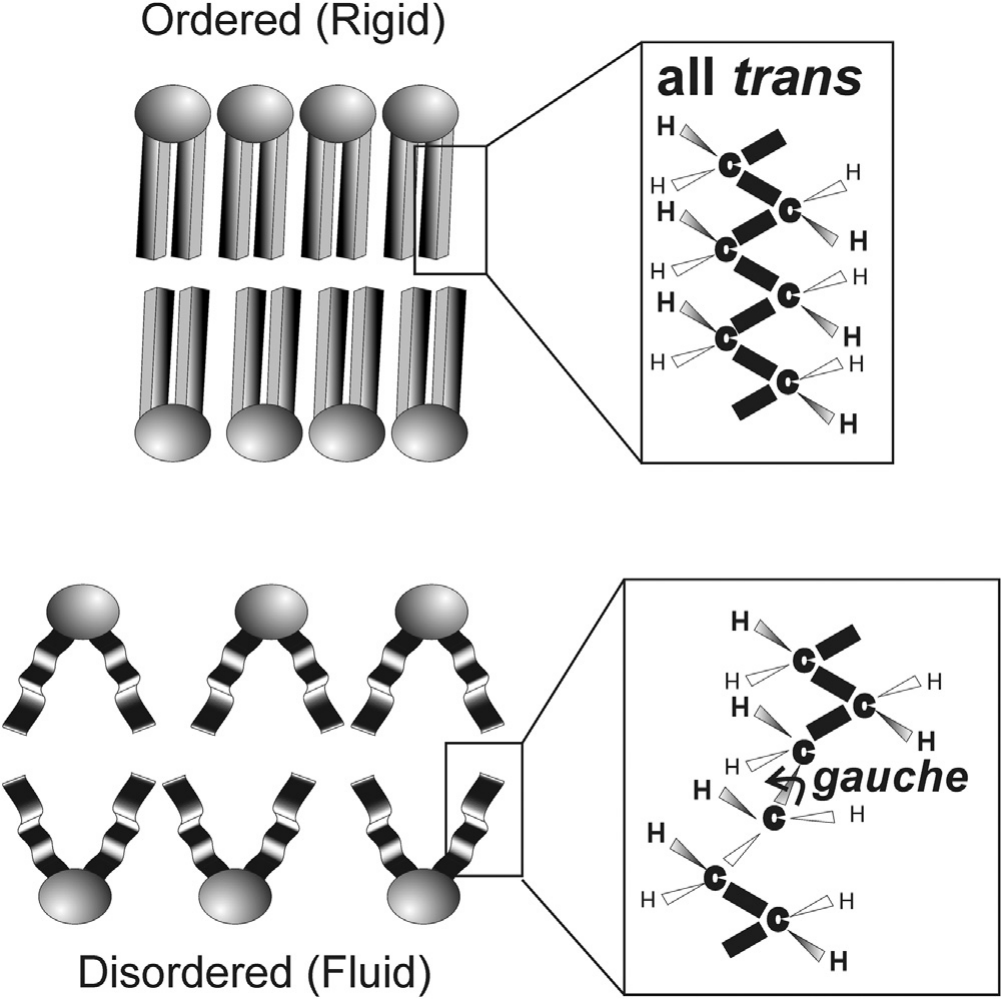
Schematic of phospholipids and the conformation of hydrocarbon chains that define lipid order. The more *trans* rotamers, the tighter the packing, the greater the van der Waal’s interactions between lipids, and the greater the lipid order (stiffness). The opposite is true for *gauche* rotamers. From (3).

**Fig. 6:**
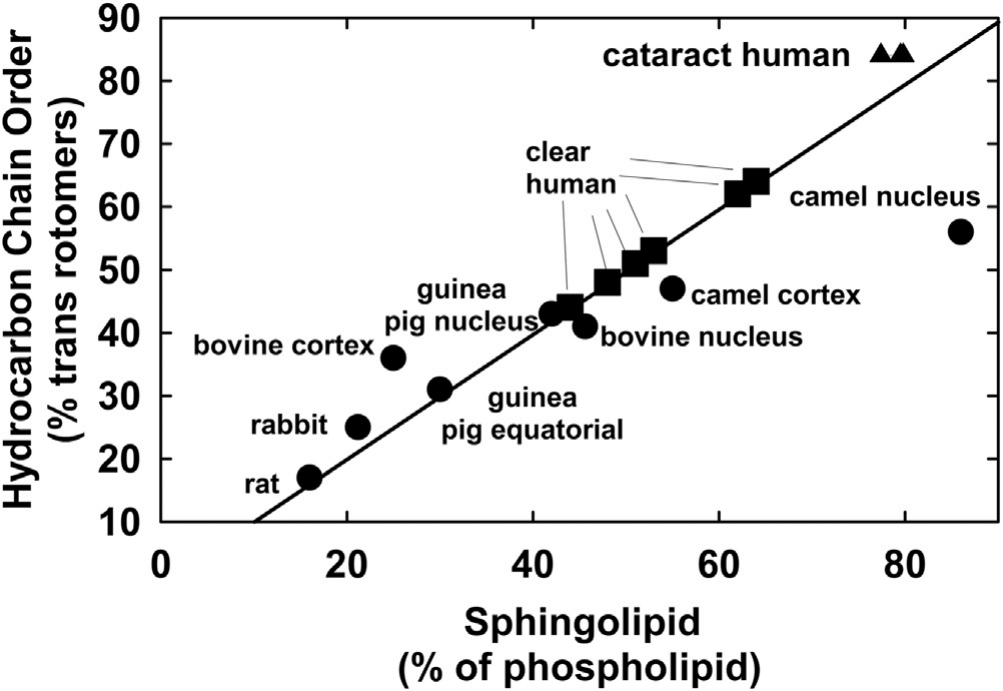
The relationship between lens sphingolipid content and hydrocarbon chain order. Hydrocarbon chain order reflects the structural stiffness of the hydrocarbon chain region of lipids in membranes. Clear human lens cortex and nucleus (closed square); cataractous human lenses (closed triangle). This figure was adapted from Figure 5 in (43). All the data except those related to cataractous lens lipid are from Borchman, Yappert, and Afzal (42). Cataractous lipid order information is extracted from Paterson et al. (45).

Hydrocarbon chain conformation may be used to measure hydrocarbon structural order, a static measure of lipid fluidity. Conformation is the spatial arrangement of atoms in a molecule that can come about through the rotation of the atoms about a chemical bond. When lipids are completely ordered, the hydrocarbons are arranged in an all-*trans* conformation of rotamers (Fig. 5, top). This allows the lipid hydrocarbon chains to pack tightly together maximizing van der Waal’s interactions between chains. When the hydrocarbon chains are disordered, the number of *gauche* rotamers increases, the lipids pack more loosely, and van der Waal’s interactions are minimal (Fig. 5, bottom). Most membranes are disordered; however, lens membranes are exceptionally ordered and the degree of lipid order increases linearly with sphingolipid content (42) (Fig. 6) and increases in the human lens with age (43) and cataract (44, 45, 46). The relationships between lens membrane order and lens clarity are discussed later in this article.

## Structure/conformation of the TFLL

**Fig. 7:**
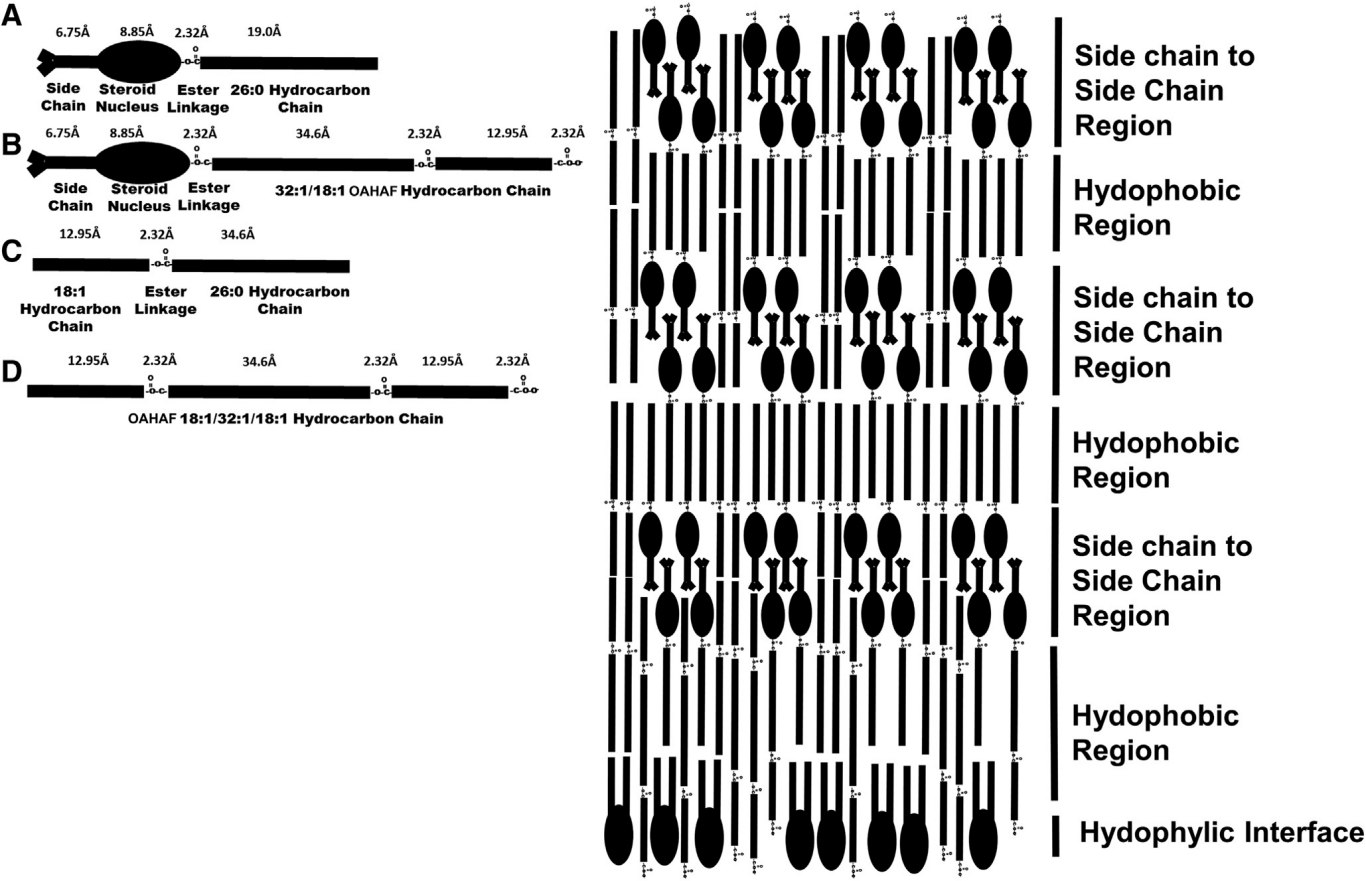
Schematic of WE and CE packing from X-ray crystallography. A: Molecular size of CE and WE with 22 carbon hydrocarbon chains. B:) Potential lamellar packing of WE. B (top): Shows rhombic packing of the hydrocarbon chains. B (right): The *trans* orientation for ordered hydrocarbons, *gauche* rotamer orientations for disordered hydrocarbon chains. C: Smectic phase packing of CE. D: Speculative packing of a WE, CE, and phospholipid mixture on an aqueous surface. From (68).

Like lens lipids discussed above, tear film lipid structural order has been measured by quantifying hydrocarbon chain conformation and structure using infrared (31, 47, 48, 49, 50, 51, 52, 53, 54, 55, 56, 57, 58, 59, 60, 61, 62, 63, 64), Raman (65), Brewster’s angle (34, 62, 66), and fluorescence anisotropy (50) spectroscopies. Meibum lipid hydrocarbons align to maximize van der Waal’s interactions between chains. Since the seminal model proposed for the packing of the TFLL in 1997 (67), a revised model has been proposed based on X-ray crystallographic studies of pure WEs and CEs (Fig. 7) (68). For a 100 nm-thick TFLL, the structure of the bulk lipids above the phospholipid monolayer consisting of esters would stack 16 times with a repeating motif. Note that the hydrocarbon chains of the WEs and CEs are not randomly oriented, as they are in an oil phase as many schematic pictures in literature show them to be. Meibum exists in a liquid crystalline phase at lower temperatures. The term “liquid crystalline phase” is used because meibum is never a solid with 100% *trans*, as the meibum hydrocarbon chains contain about 72% *trans* rotamers allowing them to pack tightly together (Fig. 5, top) (3). Thus, the term liquid crystalline phase is used rather than “solid phase”. At higher temperatures, meibum is in the gel phase and the conformation of the meibum lipid hydrocarbon chains is about 18% *trans* rotamers and 82% *gauche* rotamers (Fig. 5, bottom). Thus, meibum is not a liquid (0% *trans*) but rather in the “gel phase”. It is unknown how mixtures of WE and CE pack together, but based on X-ray crystallographic studies of pure WE and CE, it is reasonable that the minimal energy structure of the lipids is maintained in a mixture of the two (68). Thus, the lipids are shown to align to maximize hydrocarbon chain interactions (Fig. 7). Below the phase transition temperature, meibum lipids pack in an orthorhombic geometry, whereas above the phase transition temperature, the meibum lipids pack in a monoclinic geometry (54). The arrangement of molecules in Fig. 7 allows for the interdigitation of CE side chains and maximizes the adjacent packing of the steroid nuclei and CE carbonyl moieties, as they are for pure CE. Phospholipids are shown with their hydrophilic head group facing the tear aqueous layer. Phospholipids do not interact with CE (69, 70, 71); so, they are likely to form a monolayer alone with other amphipathic molecules such as (*O*-acyl)-ω-hydroxy fatty acids. A limitation of the model is that proteins, especially mucin, are likely to associate with the TFLL, but are not included in the model (72).

## Relationships between lens membrane lipid composition, structure, and function and the etiology of cataract

Human lenses differ significantly from animal lenses in regard to compaction and oxidation with age, UV filters, protein and crystallin content, synthesis of ascorbate, and antioxidant enzymes, prompting an author to state: “Unfortunately, due to marked variability in the lenses of different species, there appears at present to be no ideal animal model system for studying human ARN cataract” (Ref. (73); p. 709). Indeed, even the lens phospholipid content of numerous species, such as chickens, cows, elephants, guinea pigs, pigs, sheep, mice, and rats, varies greatly between species [see references in (43, 74, 75)]. Nevertheless, spectroscopic studies on rat, porcine, and bovine lenses have provided insights into the contribution of glucose (76), glucocorticoids (77), and cholesterol (78) on light scattering and membrane structure. Guinea pig hyperbaric oxygen (79) and UV light (80) as well as numerous other model systems have been reviewed and provided insights into the contribution of numerous factors related to human cataractogenesis that would be difficult to obtain from human lenses (81, 82, 83, 84, 85, 86).

Interspecies comparison of lens lipid composition and structure has provided insights into factors related to the etiology of cataracts. Short-lived species such as rats have a very low lens sphingomyelin content and a very high phosphatidylcholine content, whereas humans and whales that live much longer have a high lens sphingolipid content and a low lens phosphatidylcholine content. One explanation of why rats get cataracts at 2 years, dogs at 8 years, humans at 60 years, and whales do not get cataracts even after living 200 years comes from studies of lens lipid compositional and structural differences with age and species (3, 13, 42, 74, 75). At the heart of the explanation is oxidation from reactive oxidative species (87, 88), likely to originate from the mitochondria (89, 90, 91). Oxidative damage to lipids accumulates in the lens with age because there is no turnover of lipids (25) or proteins (26) disrupting crystalline structure, resulting in an increase of light scattering (92). Furthermore, products of lipid oxidation impede membrane function and alter relevant cellular processes such as growth, respiration, and ATPase and phosphate transport, as well as DNA, RNA, and protein synthesis (93). The association between lipid oxidation and lens opacity is very strong and has led many to state that lipid oxidation may be the initiating pathogen of human cataract (92, 93, 94, 95, 96, 97, 98, 99, 100). Changes in lens lipid composition with age and cataract are due to the preferential oxidation of glycerophospholipids, as explained below (3, 13, 43, 74, 75).

**Fig. 8:**
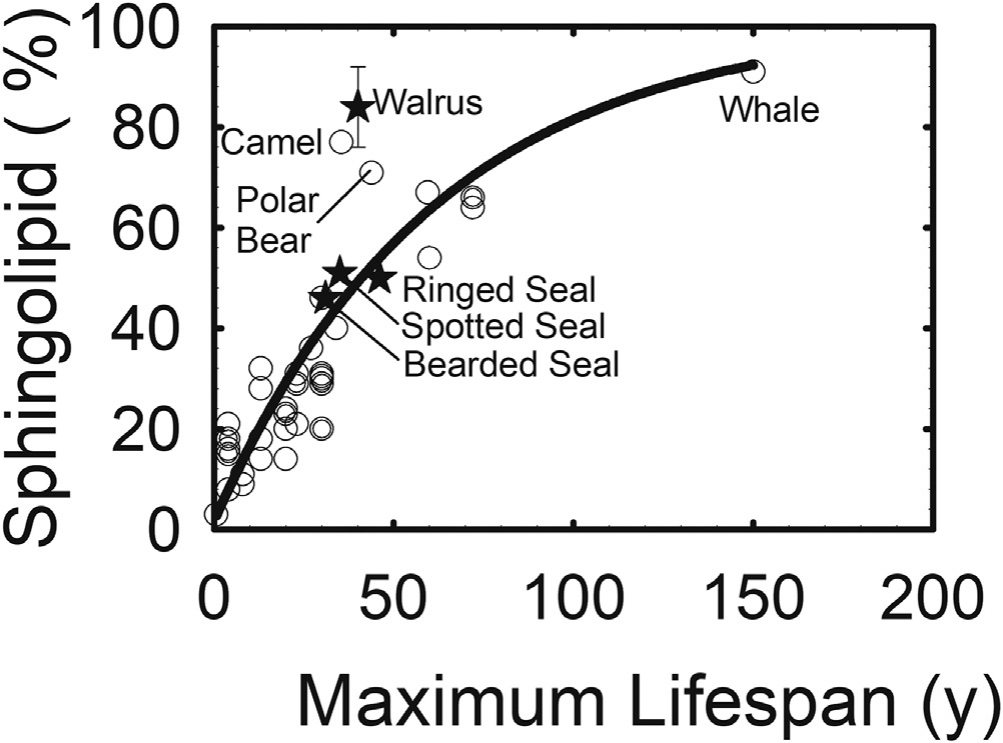
Lifespan versus lipid phase transition parameters from (74) (black stars). Data from (43, 74) (circles).

Lifespan, age, and cataract are related (3, 13, 43, 74, 75). The longer animals live, the longer they require clear lenses. Lens sphingolipid content and lifespan are correlated (Fig. 8). Does this correlation have a scientific basis or is it coincidental? Sphingolipids resist oxidation better than phospholipids (101) because they have fewer double bonds (17, 102). Sphingolipids resist oxidative degradation so much so that they were the only biomolecules found in a mammoth buried in ice for 40,000 years (103). Thus, it has been suggested that humans have adapted so that their lens membranes have a high sphingolipid content that confers resistance to oxidation, allowing their lenses to stay clear for a longer time relative to those in many other species (42). Similarly, bowhead whales (*Balaena mysticetus*) that can live over 200 years (104, 105, 106, 107, 108, 109) do not get cataracts (109), and, like humans, they have likely adapted to have the highest amount of sphingolipids in their lenses compared with other species (Fig. 8). Rats have a relatively lower level of lens sphingolipids and get cataracts relatively early, at about 2 years of age. “The strong correlation between sphingolipid and lifespan may form a basis for future studies which are needed since correlations do not necessitate cause. One could hope that if human lenses could be made to have a lipid composition similar to whales, like the bowhead whale, humans would not develop cataracts for over 100 years” (Ref. 74; p. 2289).

Diet and exposure to UV radiation are unlikely to contribute to the significant differences observed in the correlation between lifespan and phospholipid composition (Fig. 8), as rats receive less UV radiation over their lifespan and lens lipid composition does not change with extreme diets (110).

**Fig. 9:**
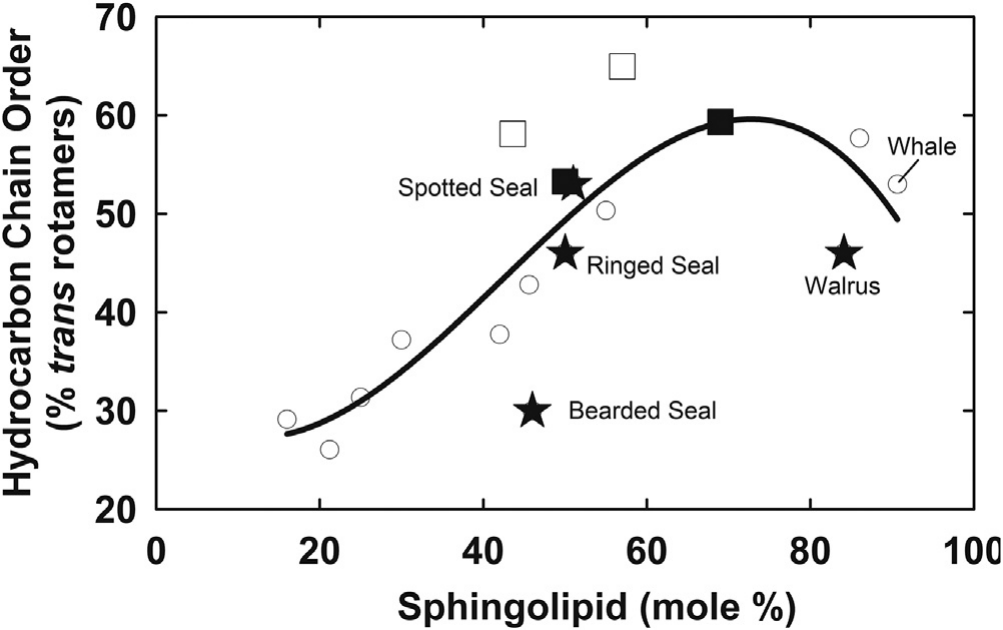
Correlation between lens sphingolipid content and hydrocarbon chain order (stiffness) from (75). Black stars, pinniped lipid [data from (43, 74, 111, 112)]; filled squares, human lens nuclear lipid; open squares, human lens cortical lipids [from (1, 95, 96)]; open circles, lenses from various species [from (75)].

In addition to the relationship between lens membrane sphingolipid content and lifespan (Fig. 8), the sphingolipid content of many animals is directly related to lipid hydrocarbon order (Fig. 9). Because of higher lipid order, membranes may be less susceptible to oxidative damage because oxygen is five times more soluble in lipid membranes than it is in the aqueous around the membrane (113, 114, 115, 116, 117, 118). In addition, oxygen is five to ten times more soluble in fluid membranes (119), such as membranes low in sphingolipids, than it is in the aqueous. It would be of value to test to determine whether organ-cultured rat lenses are more susceptible to oxidation and cataract compared with whale lenses.

In vitro studies demonstrated that ordered lipids scatter more light than disordered lipids (40). With cataract, light scattering increases by 20%, and it is speculated that the increase is due to the increase in the lipid order of lens membranes (40). It is plausible that the increase in lipid-lipid interactions may contribute to myopia by causing greater compaction and overall stiffness of the lens. In addition, lipid order influences the activity of three lens proteins, the plasma membrane and sarcoplasmic/endoplasmic reticulum Ca^2+^-ATPase activity, and aquaporin function, structure, quaternary assembly, and stability as reviewed in this journal (3).

### Factors other than hydrocarbon chain structure that relate to membrane function

**Fig. 10:**
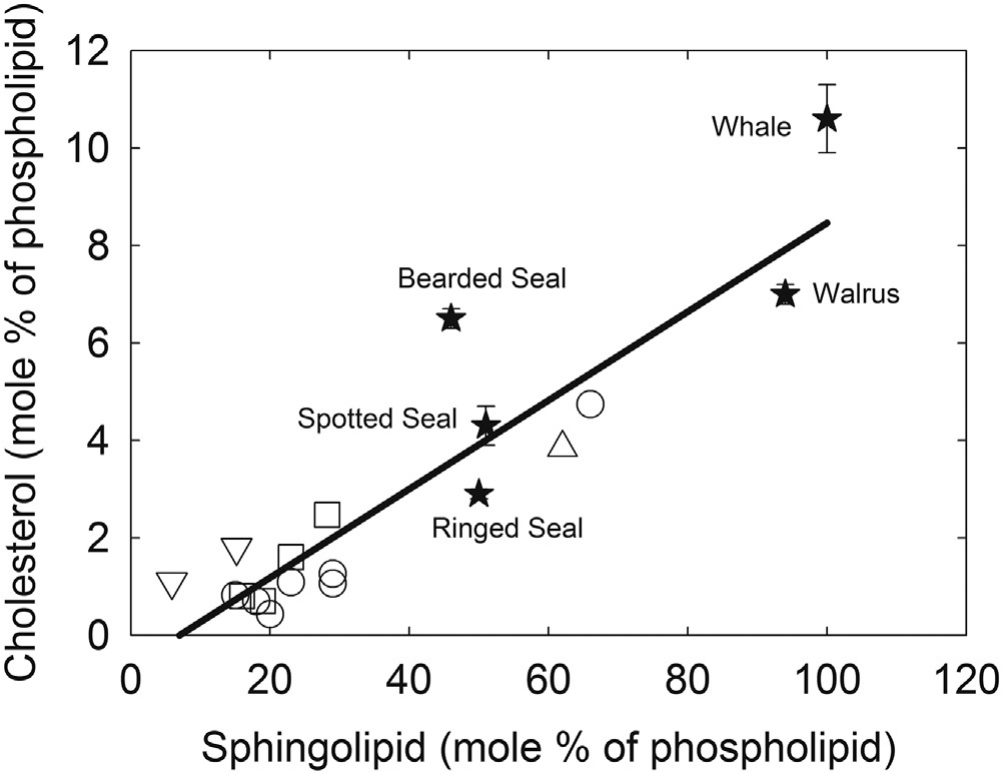
Relationship between the molar amounts of lens sphingolipid and cholesterol. Pinnipeds from (75) and bowhead whale (100% SL) from (74) (black stars). Calf lens cortex and nucleus and 2- to 6-year-old cow from (104) and 1-year-old cow from (120) (open squares). Cow, sheep, human, rat, mouse, pig, and chicken from (17) (open circles). human lens from references (37, 106) (open triangles). Mice (10 and 45 days old) from (121) (open inverted triangles). Figure from (75).

The levels of human lens sphingolipid and cholesterol are correlated (Fig. 10); however, cholesterol probably plays a minor role in determining lens membrane structure (78). It is likely to play a role in raft formation (122) and antagonize the binding of α-crystallin to lens membranes (123). It has been proposed that α-crystallin binding to lens membranes may serve as a “crystallization seed” for the binding of other proteins to the membrane, resulting in protein aggregation and light scattering (123). Thus, in addition to the inhibition of cataracts due to sphingolipids, cholesterol could inhibit protein aggregation and cataract. The relationship between cholesterol and cataract development time may also be important because cholesterol causes lens membranes to be less permeable to oxygen that may serve to keep oxygen in the outer regions of the lens long enough for the mitochondria to degrade it (117, 124).

The human (102, 103) and whale (74) lenses contain a higher amount of dihydrosphingolipid than any other organ that may inhibit lens growth by slowing the multiplication and elongation of lens cells (125). As there is no turnover of lipids (25) and proteins (26) in the lens, slow lens growth is essential in longer-lived species so that the lens does not become too large.

In summary, the long-lived species, such as humans and the bowhead whale, exhibit lens lipid adaptations that confer resistance to oxidation, thereby allowing the lens to stay clear for a relatively longer time than is the case in many other species.

## Relationships between tfll structure and function and the etiology of dry eye

### Age-related relationships between tear film stability and TFLL structure

The measurement of tear breakup time (TBUT) and blink rates (BRs), both measures of tear film stability, and TFLL structural order (see STRUCTURE/CONFORMATION OF THE TFLL above) varies greatly from person to person, complicating the analysis of correlations between these parameters. None-the-less, generalities and trends can be surmised when large cohorts are examined.

### Tear stability with age

**Fig. 11:**
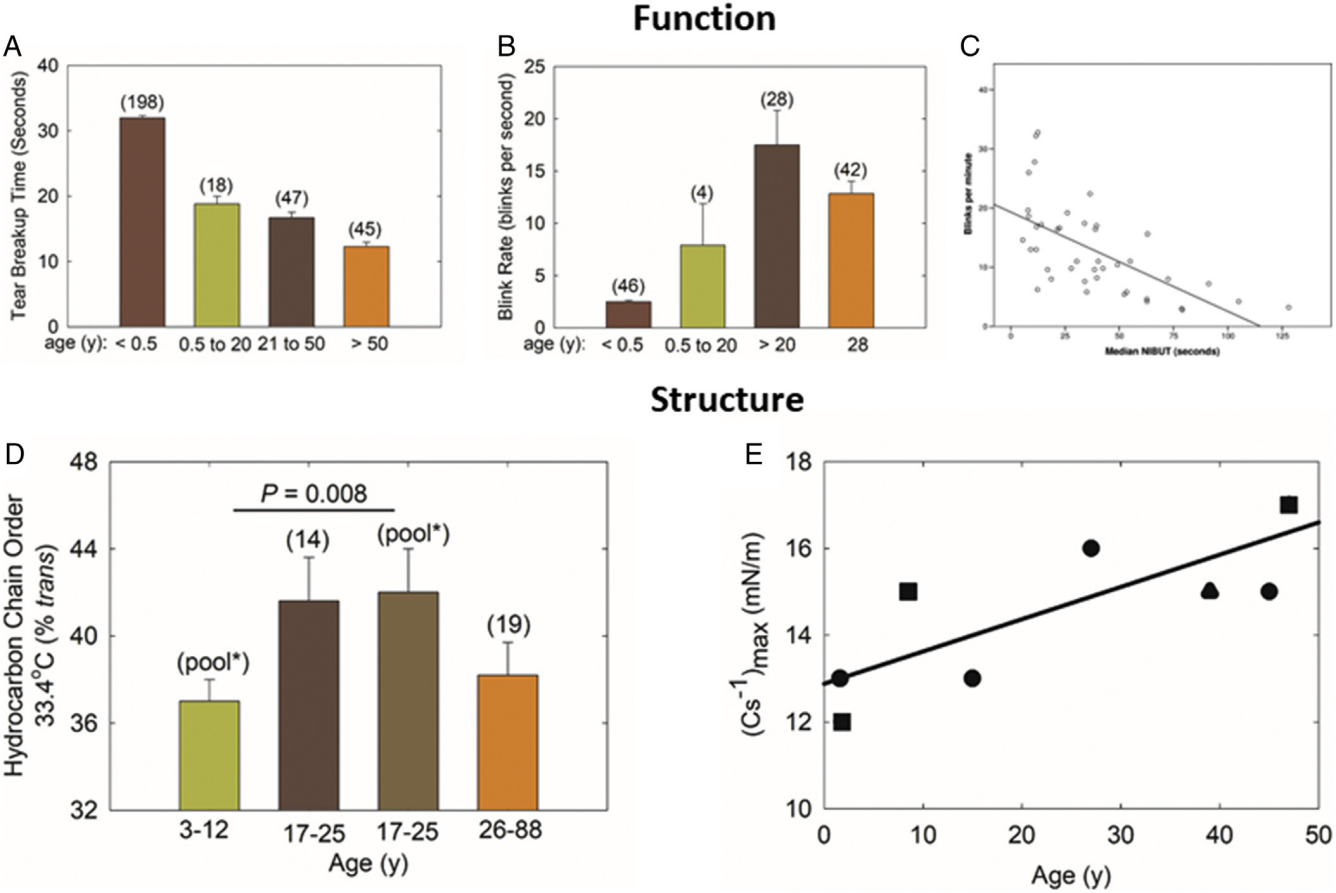
Tear breakup and BR are a measure of tear stability. Human meibum structural order was assessed by quantifying hydrocarbon chain order measured in vitro using infrared spectroscopy and (C_s_^−1^)_max_, reciprocal compressibility modulus, measured using Langmuir trough technology. A: Changes in TBUT with age. The first bar is from (126); the last three bars are from (127). B: Changes in BR with age. The first bar is from (48, 128); the middle two bars are from (48); the last bar is from (129). C: Relationship between tear film breakup time and BR from a cohort of 28 year olds (129). D: The first and third bars are from (64); the second and last bars are from (47, 50, 61). E: A larger reciprocal compressibility indicates a stiffer more elastic lipid layer. Filled circles and squares are from (130); filled triangles are from (67). Data are the average ± the standard error of the mean. The number of subjects are in parentheses.

TBUT decreases dramatically by 2.3% per year between 0.5 and 20 years of age (Fig. 11A). Between 21 and 50 years of age, TBUT decreases less dramatically by 1.03% per year, and then, above 50 years of age decreases by only 0.9% per year (Fig. 11A). Therefore, the major decline in tear film stability occurs between birth and 20 years of age.

BRs may also be used as an indirect measure of tear film stability, as BR and TBUT were inversely correlated for a cohort with an average age of 28 years (Fig. 11C) (114). Many factors contribute to BRs, such as dopaminergic activity and psychological and physiological conditions (129), but, in general, BRs reflect the level of tear film stability. Infants less than one-half of a year old blink as little as once every 2 min. The BR rises sharply between birth and 20 years of age, and then rises more gradually between 20 and 80 years of age (Fig. 11B). Therefore, like TBUT, the major increase in BR (decrease in stability) occurs between birth and 20 years of age. The slight decrease in TBUT above 50 years of age is not evident in the BR.

### Tear stability and structure

The major change in tear film stability occurs between birth and 20 years of age (Fig. 11A, B). This age-related stability change correlates with a change in the stiffness or order of the TFLL estimated from the inverse of the in-plane elasticity modulus (C_s_^−1^), also called the reciprocal compressibility modulus (Fig. 11E) (130). (C_s_^−1^)_max_ is measured using Langmuir trough technology. The (C_s_^−1^)_max_ for human meibum was measured in vitro using Langmuir trough technology and found to increase with age up to 50 years (Fig. 11E), indicating that with age, the TFLL becomes more elastic and stiff as tear film stability decreases (Fig. 11A, B) (129).

Like (C_s_^−1^)_max_ measurements, meibum structural order measured using infrared spectroscopy also shows that meibum hydrocarbon chains become more ordered (stiff) between birth and 25 years of age (Fig. 11D) (46, 50, 61, 64). Individual meibum order measurements were similar to measurements from pooled meibum, in agreement with a previous study (60). Stiffer meibum lipid hydrocarbon chains with dry eye, discussed in the next section, are also associated with a decrease in tear film stability.

One must note that changes in meibum hydrocarbon chain structural order measured using infrared spectroscopy are not always related to the elasticity or stiffness, (C_s_^−1^)_max_, measured in rheological studies. For instance, meibum and tear lipid saturation causes an increase in both the (C_s_^−1^)_max_ of tear lipid (62) and meibum (66) and the phase transition temperature of tear lipid (62) and meibum (48, 58). However, as the phase transition increases above 33.4°C, the lipid order at 33.4°C reaches a maximum value of about 80% ordered and does not change further with saturation. Thus, elasticity and meibum hydrocarbon chain order are not necessarily related when meibum structural order is high.

Like stability measurements, meibum structural order varies greatly from person to person, and large sample sizes (greater than 10) are necessary to make meaningful correlations. For instance, it was pointed out that two of seven meibum samples, 3 and 4 years of age, had an order of 62% and 67%, two standard deviation units above samples of a similar age (64). Thus, generalities made from a few samples should be made cautiously, and more measurements are needed to be certain of the structural order of meibum from babies and infants.

Above 20 years of age, the slight 1% decrease per year in TBUT is not reflected in the BR that does not change above 20 years of age (Fig. 11A, B). Perhaps this is because the BR is associated with factors such as dopaminergic activity and psychological and physiological conditions, and TBUT is not (129). Paradoxically, the slight 0.9% decline with age in TBUT above 50 years of age is associated with a decrease in meibum hydrocarbon chain order, not with an increase in age, as observed between birth and 20 years of age and in dry eye, as discussed in the next section. Perhaps hydrocarbon structural order drives the large decrease in tear stability with age between birth and adulthood, and one may speculate that above 50 years of age, TFLL compositional and macromolecular structural changes drive the decrease in tear film stability with age that is associated with a decrease in hydrocarbon chain order. (C_s_^−1^)_max_ measurements on meibum from donors above 50 years old are needed to confirm and make further correlations between tear stability and TFLL elasticity and meibum structural order for individuals older than 50 years.

In conclusion, the major decline in tear film stability between birth and 20 years of age measured by BRs and TBUT occurs concomitantly with an increase in elasticity and meibum hydrocarbon chain order measured by Langmuir trough technology and infrared spectroscopy, respectively. This correlation fits with the correlation between hydrocarbon chain order and dry eye discussed below. The relationships between elasticity and hydrocarbon structural order above 50 years of age is less clear. The functional consequences of a stiff TFLL and ordered meibum are discussed in the section below.

### Disease-related relationships between tear film stability and TFLL structure

**Fig. 12:**
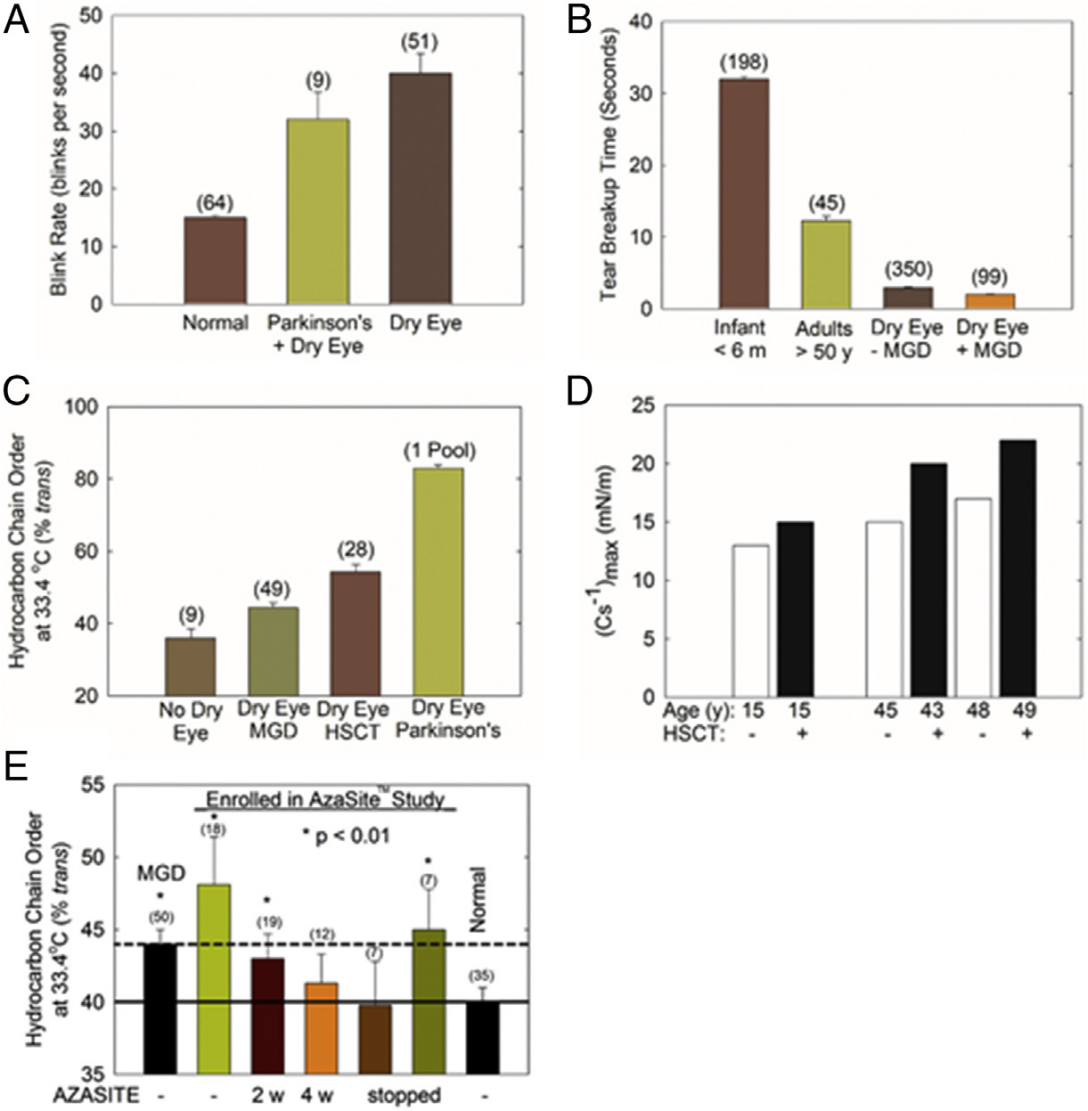
Structural functional relationships and dry eye. Tear breakup and BR are a measure of tear stability. Human meibum structural order was assessed by quantifying hydrocarbon chain order measured in vitro using infrared spectroscopy and (C_s_^−1^)_max_, the reciprocal compressibility modulus, measured using Langmuir trough technology. A: Tear film stability measured by the BR. The first and last bars are from (131); the middle bar is from (132) and is for Parkinson’s patients at stages 1 and 2 receiving dopamine agonist therapy. B: Tear film stability measured by noninvasive TBUT (NTBUT). The first bar is from (126); the second bar is from (127); the last two bars are from (133). C: Hydrocarbon chain order, a measure of lipid stiffness using infrared spectroscopy. The first bar is from (47, 50, 56) for donors 68 ± 8 years old; the second bar is from (63, 128) for donors 66 ± 6 years old; the third bar is from (41, 58) for donors 54 ± 2 years old; the last bar is from (55) for donors 66 ± 10 years old. D: Reciprocal compressibility modulus, a measure of TFLL elasticity or stiffness (130). E: A pilot study showing how when dry eye symptoms are ameliorated with treatment, lipid order is restored (51). Data are the average ± the standard error of the mean. The number of subjects is in parentheses. MGD, Meibomian gland dysfunction; HSCT, dry eye associated with hematopoietic stem cell transplantation.

Tear film instability (Fig. 12A, B) is related to elevated hydrocarbon chain order (Fig. 12C) and elevated TFLL elasticity or stiffness (Fig. 12D) with dry eye. As with age (Fig. 11A), tear film stability measured by TBUT decreases with dry eye symptoms (Fig. 12B) (126, 127, 133). TBUT and dry eye have recently been reviewed (134). Similarly, as TBUT and BR are inversely related (Fig. 11C) (114), tear film stability measured by BR also decreases with dry eye (Fig. 12A) (131). Patients with stages 1 and 2 of Parkinson’s disease who were treated with dopamine agonist therapy and are susceptible to dry eye also have high BRs (Fig. 12A) (132). As with aging (see the section above), hydrocarbon chain order is higher with dry eye due to Meibomian gland dysfunction (54, 63), dry eye in patients after hematopoietic stem cell transplantation (47, 63), and dry eye associated with Parkinson’s disease (61) (Fig. 12C). The TFLL of meibum from patients with dry eye associated with hemopoietic stem cell transplantation was more elastic and stiff, having a higher (C_s_^−1^)_max_ (Fig. 12D) compared with normal age-matched controls, in agreement with hydrocarbon chain order measured using infrared spectroscopy (Fig. 12C). Thus, hydrocarbon chain order and meibum and TFLL stiffness were related to a decrease in tear film stability. Correlation does not necessitate cause, but the relationship between hydrocarbon chain order and tear film stability is intriguing. When tear film stability is restored with treatment, lipid order is also restored to normal levels (Fig. 12E) (56), suggesting that the relationship between lipid order and tear film stability may be more than coincidental. It is reasonable to speculate that more ordered lipid could inhibit the flow of meibum from the Meibomian glands and contribute to the formation of a discontinuous patchy TFLL, which in turn results in deteriorated spreading and decreased surface elasticity (9). Indeed, one of the earliest meibum surface film studies performed in 1969 demonstrated that more ordered meibum does not spread (135), and that ordered meibum has a high surface tension resulting in poor spreading (136, 137). One may also speculate that more ordered lipid results in the attenuated capability to restore TFLL structure between blinks.

A warm compress on the eyelid is one of the oldest successful therapies to treat dry eye (63). The phase transitional parameters of meibum lipids have been used to estimate the ideal temperature needed to fluidize meibum in the eyelid and yet be safe (63). For dry eye due to Meibomian gland dysfunction, heating the eye to 41.5°C safely fluidizes meibum by 90%. For dry eye due to hematopoietic stem cell transplantation, an unsafe temperature of 52°C is needed to fluidize meibum by 90%, suggesting that other therapies are needed (63).

### Animal model studies

Most of the studies discussed in the current review involve the spectroscopy of human meibum. It is worth mentioning that, like the lens studies discussed above, insights into the etiology of dry eye can be gained from animal models that often cannot be obtained from humans. Animal models for dry eye have been reviewed (138). Antioxidants have been tested in rabbits (139). Mucins (140) and dendritic cells (141) have been studied, and a lacrimal gland excision model was developed in mice (142). TRP channels were explored (143) and artificial tears have been tested in rats (144). It has been suggested that a good animal model for human dry eye should have a similar meibum composition (145), surface properties, and BRs (146, 147). Lipidomics (145), Brewster angle spectroscopy, and surface rheology (146, 147) suggest that mice, cats, and canines meet that criterion. However, much can be learned from animal models with vastly different meibum compositions compared with humans. For instance, the tree shrew has extremely stable tears and blinks less than one time per minute (148). Is the stability of the tree shrew’s tears due to the observed longer hydrocarbon chain lengths of the tree shrew compared with human meibum lipids (152)? Koalas have extremely stable tears and can go without blinking for over 10 min (149). Could the unusual surface properties and tear film thickness measured using Brewster angle spectroscopy (149) contribute to the stability of Koalas? Future spectroscopic studies could address the question: If humans had meibum with longer chain lengths, like the tree shrew, or the unique surface properties of koalas, could humans have more stable tears?

## Relationships between TFLL composition and structure

Since the seminal studies of meibum composition in the 1970s (150, 151) and ’80s (152), many techniques have been applied to separate and quantify human tear lipid and meibum moieties such as: thin-layer chromatography, high-pressure liquid chromatography, gas chromatography, and many spectrometric techniques (18, 67, 150, 151, 152, 153, 154, 155, 156, 157, 158, 159, 160, 161, 162, 163, 164, 165, 166, 167, 168, 169, 170, 171, 172, 173, 174, 175, 176, 177, 178, 179, 180, 181, 182, 183, 184, 185, 186, 187, 188, 189, 190). The focus of the current review is on relationships between meibum and TFLL composition and structure using spectroscopic techniques such as Raman, infrared, and NMR spectroscopies. Spectrometric and other lipidomic techniques have been reviewed and were not reviewed in this article (4, 5, 6, 7, 8, 9, 10, 11, 12, 13). The relationships between meibum and TFLL composition and structure are less certain than the relationships between structure and function discussed in the previous sections. The interaction of the many lipid moieties contributes to molecular structure. For instance, as discussed in the sections below, unsaturation is a major factor that contributes to hydrocarbon chain order, yet 5 mol% saturated and ordered cholesterol ester can increase the phase transition temperature of completely disordered and unsaturated WE by an extraordinary 63°C (191). None-the-less, insights can be obtained from the spectroscopic studies discussed below. Specifically, the current review will focus on saturation, CE/WE content, hydrocarbon chain branching, and protein and squalene content and their potential contribution to meibum and TFLL structure.

### Advantages and disadvantages of using a spectroscopic approach to measure meibum and tear lipid composition

In addition to providing hydrocarbon chain conformational and structural information as discussed in the section above, infrared, NMR fluorescence, and Raman spectroscopies have been applied to the study meibum and tear lipid composition (34, 51, 53, 60, 65, 67, 68, 163, 192, 193, 194, 195, 196, 197, 198, 199, 200, 201) and as a diagnostic tool (56, 57, 195, 202, 203, 204). Raman spectroscopic studies indicate that meibum lipids are modified in the central duct, suggesting postprocessing of the lipid within the ductal region of the gland (198, 199, 200, 201, 203). The advantage and power of spectrometric lipidomic techniques is their ability to identify and quantify specific tear and meibum lipid moieties, a major advantage over using spectroscopic techniques. However, complete quantification using spectrometric techniques is complicated by the great number [thousands of species present in human meibum (152)]. Lack of specificity of spectroscopic techniques can be an advantage, especially as a diagnostic and screening tool (56, 57, 195). For instance, all the WEs have a ^1^H-NMR resonance at 4 ppm, all cholesterol resonances have a resonance at 4.6 ppm regardless of the hydrocarbon chain length, unsaturation, and branching. To measure the molar ratio of CE to WE using NMR spectroscopy, one only needs to divide the ratio of the intensity of the CE resonance by the intensity of the WE resonance. The molar ratio of CE to WE can be calculated without the need to know the molecular weights of the moieties by accounting for the stoichiometry of the hydrogen resonances, 2 mol from the WE and 1 mol from the CE. To measure the CE to WE molar ratio by spectrometric measurements, one must identify and quantify all of the hundreds of species that are associated with CE and compare them to the sum of the hundreds of species that were identified as WE. Because each lipid class has a different ionization efficiency or response factor, standards are needed for the spectrometric quantification of each of the thousands of moieties in meibum. As stated above, standards are not needed for quantification using NMR spectroscopy. Furthermore, the sample is not destroyed using spectroscopic measurements, so the same sample could be used for spectrometric measurements, other spectroscopic techniques, or rheological studies.

**Fig. 13:**
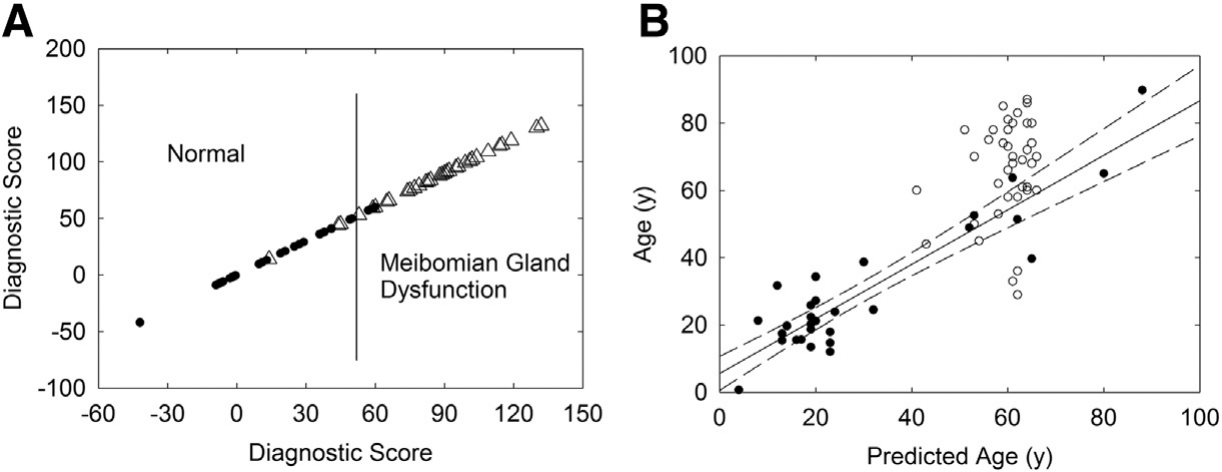
A: A training set was used to discriminate between the spectra of meibum from normal donors and the spectra of meibum from donors with meibomian gland dysfunction. This shows that the infrared spectra must contain compositional and structural information about the changes that occur with meibomian gland dysfunction. A score above 50 (vertical line) is considered a sample with meibomian gland dysfunction. B: The same training set used for A was used to predict the age of the donors with meibomian gland dysfunction within ±20 years at a 95% confidence limits. M_n_ group (filled circles); Md group (open circles); linear regression fit (solid line); and 95% confidence limits of M_n_ group (dashed lines). From (53).

Principal component analysis of infrared and NMR spectra of meibum has been used to discriminate and diagnose the “principal component,” Meibomian gland disfunction or age, with an accuracy of greater than 93% (Fig. 13) (51, 53, 195). More importantly, eigenvectors created from variations in spectra associated with the principal component contain information about the changes that occur in the composition and structure of meibum. Principal component analysis is not subjective, and the spectroscopic expertise needed is minimal for the diagnosis and screening of samples.

### Hydrocarbon chain saturation

**Fig. 14:**
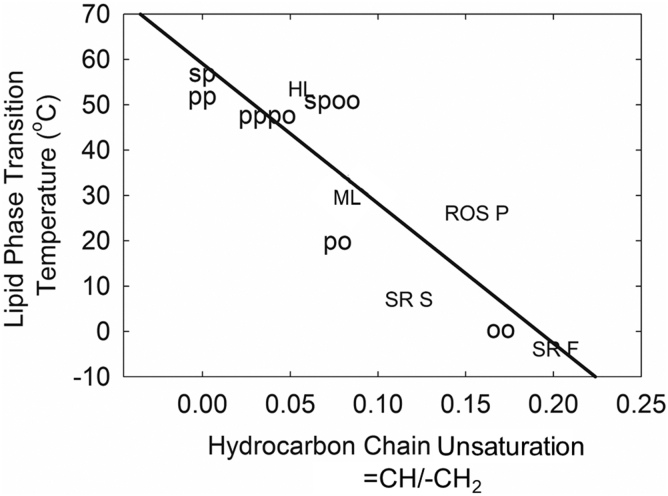
Relationship between phase transition temperature and hydrocarbon chain saturation. Samples measured in this study: oo, oleyloleate; po, palmityloleate; pp, palmitylpalmitate; sp, sterylpalmitate; pppo, equal molar mixture of pp and po; spoo, equal molar mixture of sp and oo. HL, human lens lipid; ROS P, bovine rod outer segment plasma membrane,;SR F, fast twitch rabbit muscle sarcoplasmic reticulum membrane; SR S, slow twitch rabbit muscle sarcoplasmic reticulum membrane. Least squares linear regression fit to all of the data is denoted by the diagonal line. From (58).

**Fig. 15:**
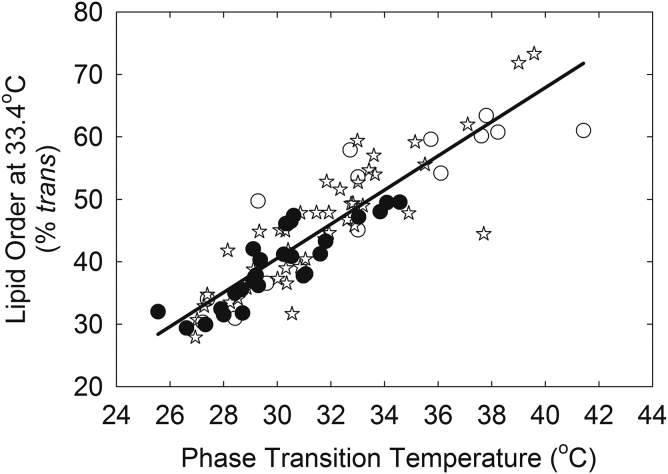
Correlation between the lipid phase transition temperature and lipid order at 33.4°C for human meibum. Meibum from donors without dry eye (filled circles); meibum from donors with dry eye and hematopoietic stem cell transplantation (open circles); meibum from donors with meibomian gland dysfunction (open stars). From (47).

It is well established that hydrocarbon chain saturation is the major factor that contributes to the phase transition temperature of native and synthetic lipids (Fig. 14) (58). The phase transition of human meibum is about midway between that of the unsaturated disordered WE, oleyl oleate, and the saturated ordered wax, palmityl palmitate. Because the phase transition of meibum is near physiological temperature and because the phase transition temperature and lipid hydrocarbon chain conformational order are closely related (Fig. 15), small changes in the phase transition temperature can cause significant changes in hydrocarbon chain order. The effect of catalytic saturation on the surface properties of human meibum and tear lipids was studied using Langmuir trough, and infrared and Brewster’s angle spectroscopies, respectively (48, 58, 62, 66). The increase in the percentage of saturation resulted in thicker and more elastic meibum and tear lipid films with the effects being proportional to the saturation level. Saturation concomitantly increased the phase transition temperature, hydrocarbon chain order, phase transition cooperativity, and the change in entropy and enthalpy. Native tear lipids were more ordered and elastic compared with meibum lipids collected from the same individual (62). Aggregation of lipids on the tear surface due to saturation was not as significant as it was for meibum (62).

Principal component analysis of meibum infrared spectra indicated that lipid saturation is higher in meibum from donors with dry eye due to Meibomian gland dysfunction that could account for elevated lipid order (53). However, principal component analysis is qualitative, and Raman (65), NMR (194), and an infrared spectroscopic study (60) show that there is no difference in saturation for meibum from donors with dry eye due to Meibomian gland dysfunction (M_MGD_) or without Meibomian gland dysfunction (M_n_). Meibum (M_HSCT_) and tears from patients who had dry eye due to hematopoietic stem cell transplants were more ordered and had fewer double bonds than samples from patients without dry eye (60).

Unsaturation may contribute to the decrease in meibum lipid order above 20 years of age (48); however, a qualitative infrared study did not confirm this finding, as adolescents 16–23 years of age and adults 32–61 years of age had the same saturation levels (51). There is also no evidence that the rise in lipid order between 1 and 20 years of age is due to saturation, as meibum from children was more saturated than adolescents and adults, contrary to what would be expected (48, 51, 196). Thus, although the stiffness of meibum lipid can be largely attributed to saturation, whether or how unsaturation levels contribute to the rise in meibum order between 0 and 20 years of age and the subsequent decrease in order above 20 years of age is not clear. Perhaps the level of CE, discussed below, more closely relates to lipid order.

### CE levels

**Fig. 16:**
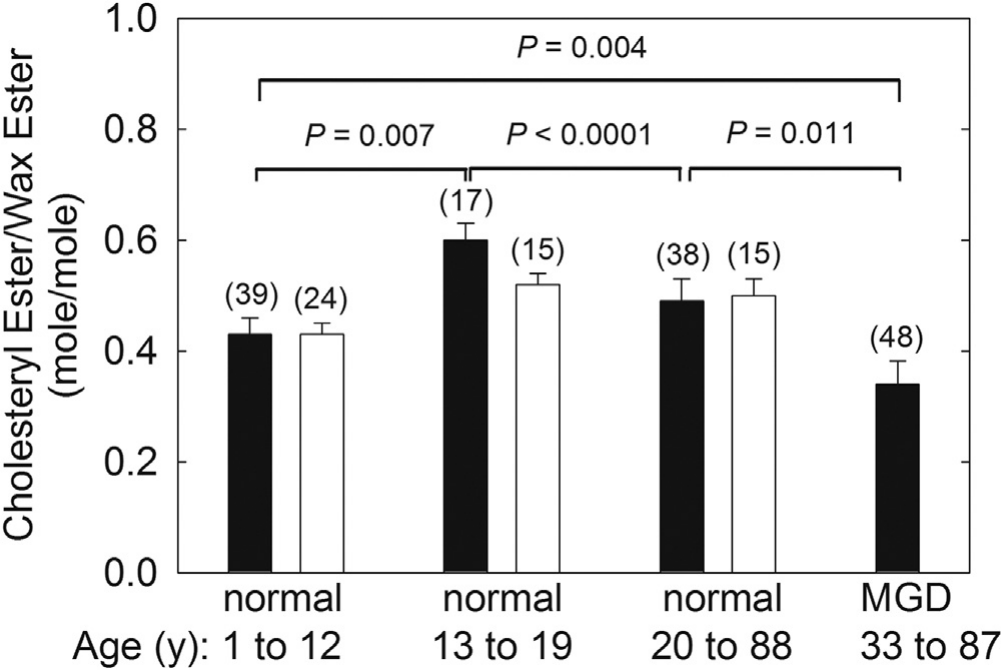
CE/WE molar ratios calculated from the NMR spectra of meibum. Solid bars: Molar ratios calculated from the intensity of the CE resonance at 4.6 ppm and the WE resonance at 4.0 ppm. Correcting for (*O*)-acylated ω-hydroxy fatty acids the solid bars would be lower. For instance, the value for Meibomian gland disfunction corrected would be 31, lower than the reported value of 34. Open bars: Molar ratios calculated from the intensity of the CE resonances from cholesteryl carbon numbers 18 and 19 and the WE resonance at 4.0 ppm. From (68).

CEs are likely to partition with WEs in the bulk TFLL (Fig. 7). NMR spectroscopy shows CE/WE molar ratios were higher in donors 13–19 years old compared with donors 1–12 years old and 20–88 years old (Fig. 16) (61), which correlates well with the changes in lipid order observed with age (Fig. 11D). It is attractive to speculate that changes in CE concentration with age cause molecular structural changes and diminished tear film stability. Model studies strengthen the idea that CE increases the order of CE-WE mixtures with age (191, 193). CE concentration from 0 to 100 mol% linearly increased the phase transition temperature of the WE-CE mixture. As little as 5 mol% saturated CE raised the phase transition temperature of the unsaturated WE, oleyl oleate, from −0.1°C to 65°C (191). CE at 50% increased the phase transition of WE by 14°C (193).

Infrared spectroscopy and NMR spectroscopy also shows that patients with dry eye due to Meibomian gland dysfunction (Fig. 16) (68, 193) and Parkinson’s disease (130) have about 70% lower cholesteryl levels in M_MGD_ compared with M_n_. Donors with Parkinson’s disease that do not have dry eye have 35% less CE in their meibum compared with M_n_, perhaps a reason why people with Parkinson’s disease are more susceptible to dry eye (unpublished observations, S. Blinchevsky, A. Ramasubramanian, D. Borchman, S. Sayied, K. Venkatasubramanian). Because the amount of CE decreases with dry eye, one would expect a decrease in lipid order in meibum from donors with dry eye, not an increase as observed (Fig. 12). It is possible that the increase in lipid order and decrease in CE is coincidental and just a marker of dry eye, that other factors lead to increased meibum order with dry eye, and that model studies with simple WE and CE do not emulate the complex structure of native meibum WE and CE. It is also possible that a lower meibum CE/WE ratio with dry eye leads to structural macromolecular packing changes in the TFLL other than molecular conformational changes in the hydrocarbon chains. It is still attractive to speculate that therapies to increase the CE/WE ratio in meibum could restore the structure and ameliorate dry eye symptoms, a speculation worth testing.

### Hydrocarbon chain branching and CH_3_ moieties

**Fig. 17:**
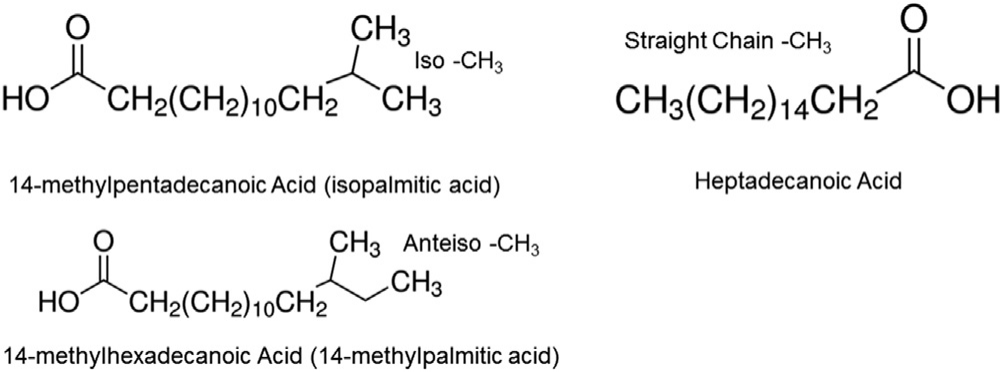
Examples of straight chain hydrocarbons and branched chain hydrocarbons. From (194).

Hydrophobic van der Waals interactions between CH_2_ moieties are responsible for the tight hydrocarbon chain packing necessary for lipid ordering (Fig. 5). CH_3_ moieties sterically inhibit CH_2_-related hydrocarbon-hydrocarbon hydrophobic interchain interactions and cause lipids to be more disordered, as observed with anteiso branching (Fig. 17) (194). The CH_3_ and CH_2_ infrared stretching bands are the most prevalent bands in the infrared spectra of meibum and tear lipids and were used to quantify the CH_3_/CH_2_ peak height ratios of M_n_, M_MGD_, and M_HSCT_ (47, 54). M_MGD_ (53, 60) and M_HSCT_ (60) had relatively fewer CH_3_ moieties compared with M_n_. As stated earlier, CH_3_ moieties cause lipids to be more disordered, so it is reasonable that fewer CH_3_ moieties could contribute to meibum from donors with dry eye and M_HSCT_ having a higher hydrocarbon chain order compared with M_n_.

Resonances for anteiso, iso, and straight chain branching were verified and quantified using the NMR spectra of human meibum (194), and resonance assignments (133) confirmed (175). M_HSCT_ contained fewer branched chains compared with M_n_, in agreement with the infrared studies above (unpublished observations, P. Mudgil, A. Ramasubramarian, D. Borchman). This supports the idea that branching could contribute to the higher hydrocarbon chain order and elasticity of M_HSCT_ compared with M_n_ (Fig. 12C, D). However, branching differences were not observed for meibum from Parkinson’s patients with or without dry eye (unpublished observations, S. Blinchevsky, A. Ramasubramanian, D. Borchman, S. Sayied, K. Venkatasubramanian). It is also unlikely that hydrocarbon chain branching contributes to the high hydrocarbon chain order for M_MGD_, as M_MGD_ contained more iso-branched hydrocarbon chains compared with M_n_ (194). This difference in branching is unlikely to contribute to hydrocarbon chain order as iso-branched lipids melt at the same temperature as straight chain lipids.

### Protein, squalene, sebum, and meibum order

The structure and function of the TFLL and meibum are complicated by proteins. Raman spectroscopy has been used to quantify proteins in tears (209) and to determine that “the highest fraction of pure lipid found in subjects with an unstable tear film” (200, p. 158) is at the orifice and decreases toward the acinus of the Meibomian glands (207, 208, 210). The amount of protein in meibum is related to the lipid order of meibum (53). Whether this relationship is coincidental or causal has yet to be investigated. Earlier studies speculated that keratins aggregate meibum and block the flow of meibum from the Meibomian glands (204, 205, 206, 207, 208). Infrared spectroscopy showed that keratin does not change the order of wax, so the mechanism of blockage does not involve the influence of keratin binding to WE, causing WE to become stiffer and aggregate (72). All the major proteins found in tears bind to the TFLL and are the major contributors to the surface pressure (209, 210, 211, 212, 213, 214). An infrared spectroscopic study showed that mucin disordered wax (72), that is perhaps why it facilitates the spreading of meibum.

About 10% of the TFLL comes from sebum (Fig. 3) (31, 32). Sebum expanded and fluidized meibum and lowered lipid order, the phase transition temperature, and cooperativity, all believed to stabilize meibum (Fig. 3) (26). Squalene, not a large component of meibum [1% (32, 33)] but a large component of sebum (20%) (192) and the TFLL (7%), has no surface properties and stabilizes the TFLL by filling in gaps (34).

The spectroscopic studies reviewed relating to how lipid composition, conformation, and function are involved in the etiology of cataract and dry eye are but a small part of the studies involving other techniques and approaches not covered by this review. A plethora of future studies are needed to understand how the vast complexity of the components in lens membranes and the TFLL interact with one another.

## Conflict of interest

The author declares that he has no conflicts of interest with the contents of this article.
